# P-988. Carbapenems on the watch: monitoring the use of carbapenem and its resistance as part of Antimicrobial Stewardship in a Caribbean teaching Hospital

**DOI:** 10.1093/ofid/ofaf695.1187

**Published:** 2026-01-11

**Authors:** Rita A Rojas-Fermín, Anel E Guzmán-Marte, Ricardo Ernesto Hernandez-Landa, Lucy Hernandez-Rosado, Cristian Tejada-Joaquin

**Affiliations:** Hospital General De La Plaza De La Salud, Distrito Nacional, Distrito Nacional, Dominican Republic; Hospital General De La Plaza De La Salud, Distrito Nacional, Distrito Nacional, Dominican Republic; Universidad Ibero Americana, Santo Domingo, Distrito Nacional, Dominican Republic; Hospital General Plaza de la Salud, santo Domingo, Distrito Nacional, Dominican Republic; Hospital General Plaza de la Salud, santo Domingo, Distrito Nacional, Dominican Republic

## Abstract

**Background:**

Antimicrobial stewardship (AMS) initiatives typically lack the funding necessary for active surveillance personnel, tools or molecular tests in countries that have limited resources. Since the link between the use of antimicrobials and its resistance is well documented, incorporating the Defined Daily dose (DDD) of antimicrobials to our AMS strategies could provide further insight to this matter. We aim to describe the relationship between DDD and CRE incidence tendencies over a two-year period in a teaching hospital in the Dominican Republic.

**Figure:**
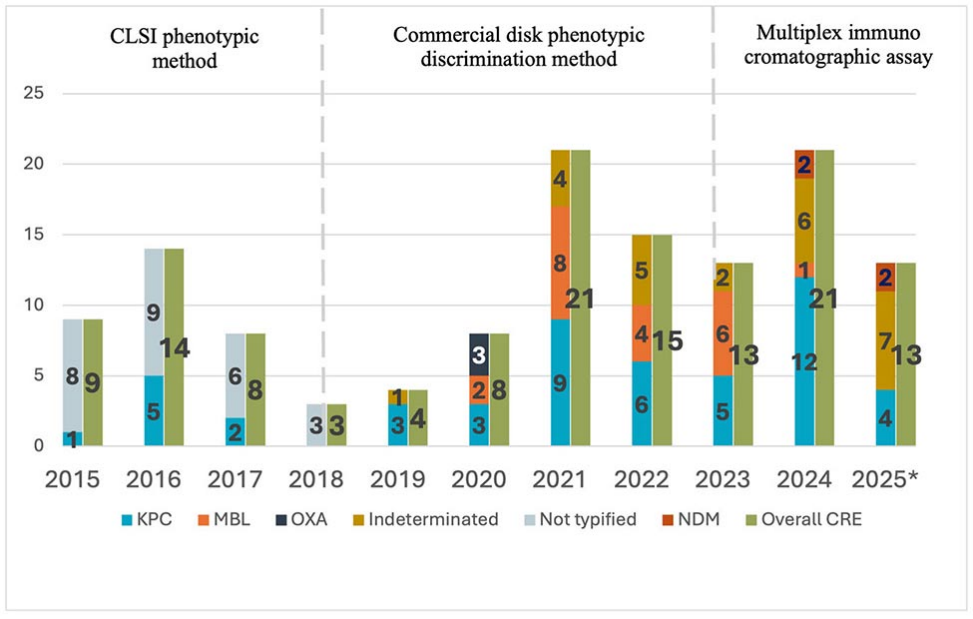
Carbapenem Resistant Enterobacterales incidence per year .

**Figure:**
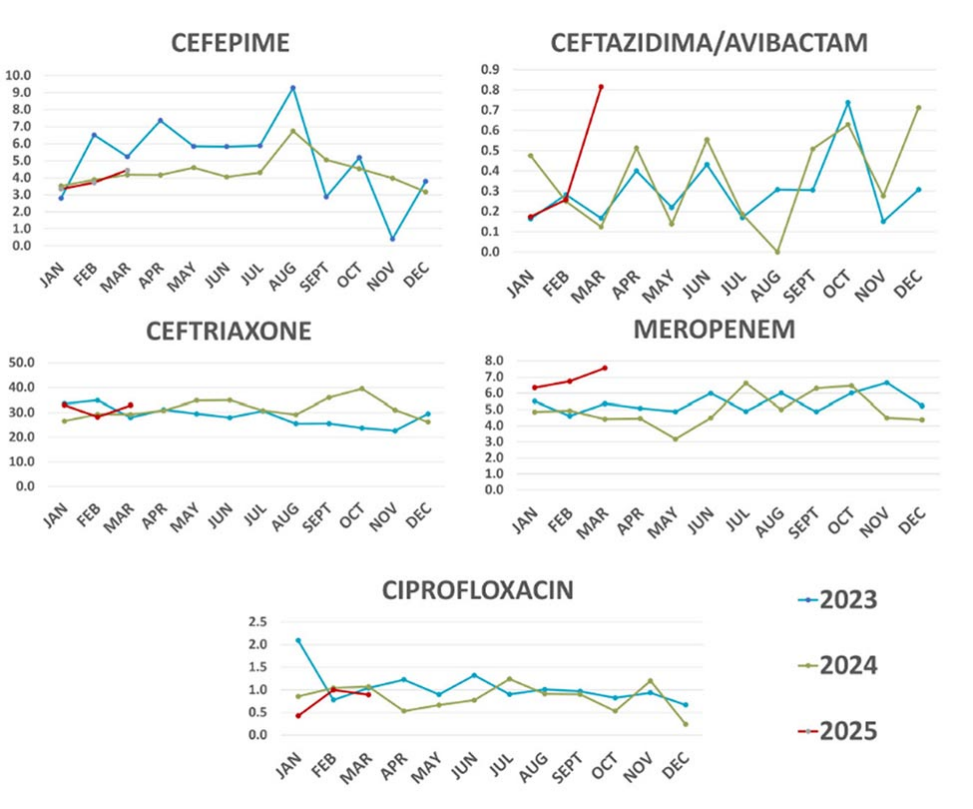
Monthly Defined Daily Dose per 100-beds

**Methods:**

We designed a retrospective cross-sectional study including monthly average DDD measures from January 1, 2023, toMarch 31, 2025. Defined Daily dose (DDD) of antimicrobials used per-100 bed-days (mean DDD/year) was tabulated.Available carbapenem-resistant Enterobacterales (CRE) frequency and their carbapenemase type was charted fromJanuary 2015 to March 2025. Both tendencies were compared to identify possible association between them.

**Results:**

KPC is the most frequent type of carbapenemase since 2015, but there has been an increasing tendency to MBL detection. The incidence of CRE to the first trimester of 2025 (1/3 of 2025) is higher than mean number of CRE isolates per year in previous years (13 vs 11.6, respectively). There is a notable increase in meropenem use in the1/3 of 2025 when compared to the first trimester of previous years, even though other antimicrobials like ciprofloxacin, cefepime and ceftriaxone have remained stable but considerably high.

**Conclusion:**

Active surveillance is necessary for real-time detection of antimicrobial use and its relationship with resistance. Carbapenem resistance has increased during the last 10 years and there is rising concern around its possible correlationwith a higher prescription of meropenem in the hospital. Record of tendencies throughout time offers better insight of the status. Infection Control measures must be taken to curb the spread of other carbapenemases such as NDM.

**Disclosures:**

Rita A. Rojas-Fermín, MD, GSK: Honoraria

